# Chloride channels are necessary for full platelet phosphatidylserine exposure and procoagulant activity

**DOI:** 10.1038/cddis.2013.495

**Published:** 2013-12-19

**Authors:** M T Harper, A W Poole

**Affiliations:** 1 School of Physiology and Pharmacology, Bristol Heart Institute, Medical Sciences Building, University of Bristol, Bristol BS8 1TD, UK

**Keywords:** necrosis, coagulation, platelets, thrombosis, chloride channels

## Abstract

Platelets enhance thrombin generation at sites of vascular injury by exposing phosphatidylserine during necrosis-like cell death. Anoctamin 6 (Ano6) is required for Ca^2+^-dependent phosphatidylserine exposure and is defective in patients with Scott syndrome, a rare bleeding disorder. Ano6 may also form Cl^−^ channels, though the role of Cl^−^ fluxes in platelet procoagulant activity has not been explored. We found that Cl^−^ channel blockers or removal of extracellular Cl^−^ inhibited agonist-induced phosphatidylserine exposure. However, this was not due to direct inhibition of Ca^2+^-dependent scrambling since Ca^2+^ ionophore-induced phosphatidylserine exposure was normal. This implies that the role of Ano6 in Ca^2+−^dependent PS exposure is likely to differ from any putative function of Ano6 as a Cl^−^ channel. Instead, Cl^−^ channel blockade inhibited agonist-induced Ca^2+^ entry. Importantly, Cl^−^ channel blockers also prevented agonist-induced membrane hyperpolarization, resulting in depolarization. We propose that Cl^−^ entry through Cl^−^ channels is required for this hyperpolarization, maintaining the driving force for Ca^2+^ entry and triggering full phosphatidylserine exposure. This demonstrates a novel role for Cl^−^ channels in controlling platelet death and procoagulant activity.

The central role of blood platelets in arterial thrombosis critically depends on their ability to promote localized thrombin generation.^1^ High, sustained Ca^2+^ signals trigger necrotic cell death in platelets resulting in phosphatidylserine (PS) exposure in the outer leaflet of the plasma membrane.^2^ Platelet PS forms a procoagulant surface that accelerates thrombin generation. It is currently believed that sustained intracellular Ca^2+^ signalling activates a nonspecific phospholipid ‘scramblase' while inhibiting an inward aminophospholipid translocase (‘flippase').

Ano6 (gene *TMEM16F*) is a key regulator of calcium-dependent PS exposure.^3^ Loss-of-function mutations in *TMEM16F* have been identified in two Scott syndrome patients,^3, 4^ who have defective calcium-dependent PS exposure in platelets, erythrocytes and B lymphocytes.^5^ However, how Ano6 contributes to PS scrambling is not clear.^6^ Several groups have shown that Ano6 acts as a Cl^−^ channel regulated by Ca^2+^ or cell volume.^7, 8, 9, 10^ However, it is not immediately clear how PS exposure could be directly regulated by Cl^−^ conductance.

In this study, we have used platelets to test whether Cl^−^ channels in principle can regulate Ca^2+^-dependent PS exposure. We find that Cl^−^ channel blockers do not affect PS exposure in response to a Ca^2+^ ionophore, implying that Cl^−^ conductances are not necessary for platelet PS exposure. However, we also found that Cl^−^ channel blockers reduce PS exposure in response to physiological platelet activators. Cl^−^ channels are required for plasma membrane hyperpolarization in stimulated platelets, which in turn is required for sustained Ca^2+^ signalling. These data demonstrate a novel role for Cl^−^ channels in platelet death and procoagulant activity.

## Results

**Figure 1:**
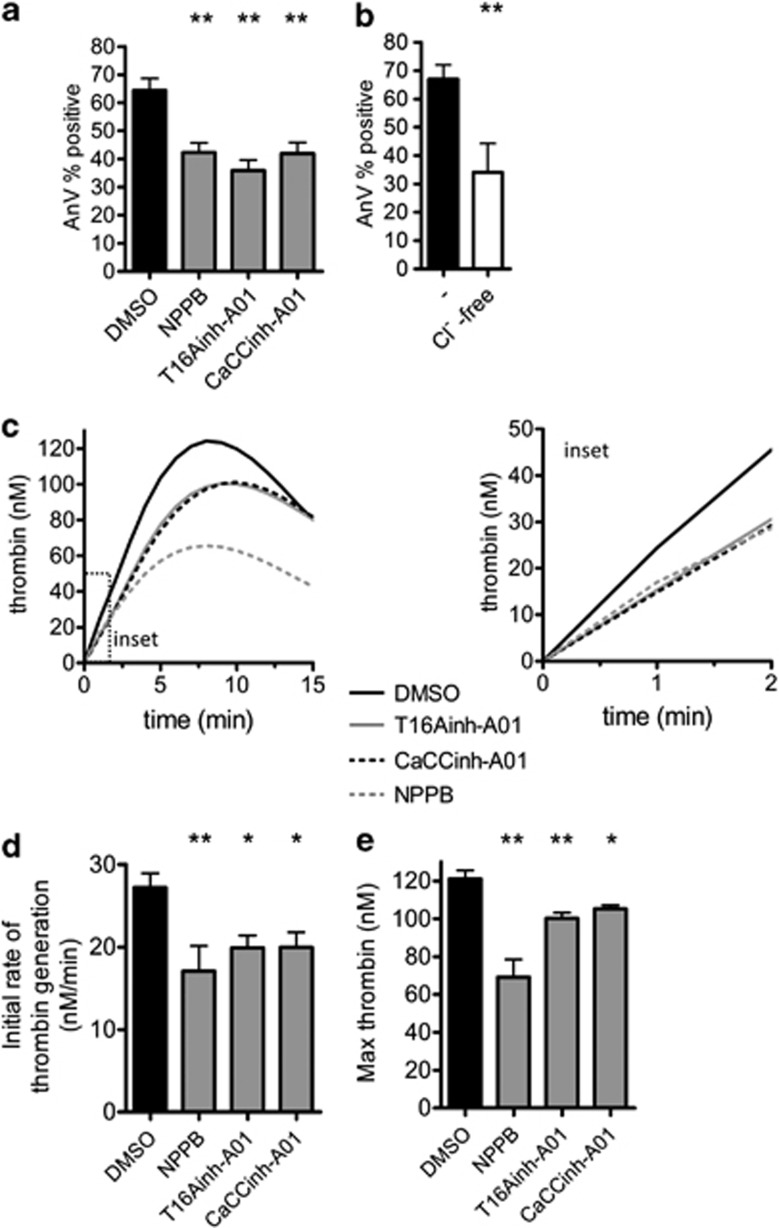
Chloride channel blockers partially inhibit the platelet procoagulant response. (**a**, **b**) Washed platelets were stimulated with thrombin (1 U/ml) and CRP (5 *μ*g/ml) for 10 min and the level of phosphatidylserine exposure determined by annexin V (AnV) binding and flow cytometry. Histograms depict mean±S.E.M. (*n*=4–9; *P*<0.01). (**a**) Platelets were treated with the indicated chloride channel blockers, or equivalent volume of DMSO as control, for 5 min before stimulation. (**b**) Platelets were suspended in normal NaCl-based buffer (–) or Na-gluconate-based buffer (Cl^−^ free). (**c**–**e**) The ability of CRP-stimulated platelets to support thrombin generation in plasma was determined using a fluorescent thrombin substrate, as described in Materials and Methods. The traces from an individual experiment (**c**) are representative of four independent experiments. The right panel shows the first two minutes. (**d**, **e**) The initial rate of thrombin generations and peak thrombin generation are quantified (*n*=4; *P<0.05; **P<0.01)

When platelets were co-stimulated by thrombin and collagen-related peptide (CRP, an agonist of GPVI (glycoprotein VI)), a substantial fraction exposed PS on their surface, as detected by annexin V binding (Figures 1a and b). To test whether Cl^−^ channels have a role in this, three structurally distinct Cl^−^ channel blockers were tested. NPPB blocks multiple Cl^−^ channels.^7, 11^ T16Ainh-A01 was identified in a screen for TMEM16A inhibitors.^12^ CaCCinh-A01 blocks Ca^2+^-activated Cl^−^ channels^13^ and TMEM16F-dependent Cl^−^ currents.^7, 14^ Each inhibitor significantly reduced thrombin-plus-CRP-induced annexin V binding (Figure 1a), indicating that Cl^−^ channels are necessary for full platelet PS exposure.

Thrombin-plus-CRP-induced PS exposure was also significantly reduced in the absence of extracellular Cl^−^ (Figure 1b; Cl^−^ replaced by equimolar gluconate). This demonstrates that the effect of the Cl^−^ channel blockers is not a nonspecific effect on other ion channels. Together, these data indicate that Cl^−^ entry through Cl^−^ channels is required for full agonist-induced PS exposure.

The role of Cl^−^ channels in thrombin generation in platelet-rich plasma was determined by the calibrated automated thrombogram (CAT) method.^15^ Platelets were stimulated with CRP, and thrombin generation was initiated with tissue factor and CaCl_2_. Pre-treatment with Cl^−^ channel blockers significantly reduced and slowed thrombin generation, indicating an important physiological role for these channels (Figures 1c–e).

**Figure 2:**
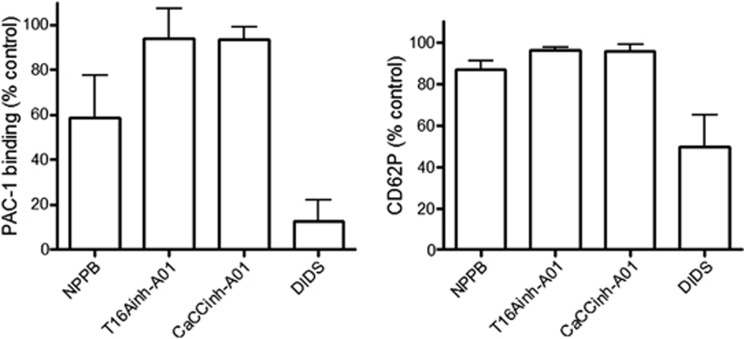
Effect of Cl^−^ channel blockers on integrin *α*_IIb_*β*_3_ activation and *α*-granule secretion. Washed platelets were treated with the indicated Cl^−^ channel blockers (or DMSO) as control then stimulated with thrombin (1 U/ml) in the presence of FITC-conjugated PAC-1 antibody (left panel) or FITC-conjugated anti-CD62P antibody (right panel). Platelet staining was determined by flow cytometry and expressed as percentage of fluorescent signal in DMSO-treated, thrombin-stimulated platelets. (*n*=3–4). Histograms depict mean±S.E.M.

The Cl^−^ channel blockers did not significantly inhibit thrombin-induced P-selectin expression (a marker of *α*-granule secretion). Thrombin-induced *α*_IIb_*β*_3_ activation was not affected by T16Ainh-A01 and CaCCinh-A01 but was partially reduced by NPPB. Another commonly used Cl^−^ channel blocker, DIDS, substantially reduced P-selectin expression and almost abolished *α*_IIb_*β*_3_ activation, suggesting that it may have substantial off-target effects, and so was not used further in this study (Figure 2). The greater effect of NPPB on peak thrombin generation may reflect its additional effect on *α*_IIb_*β*_3_ activation as signalling through *α*_IIb_*β*_3_ is reported to regulate thrombin generation.^16, 17, 18^

**Figure 3:**
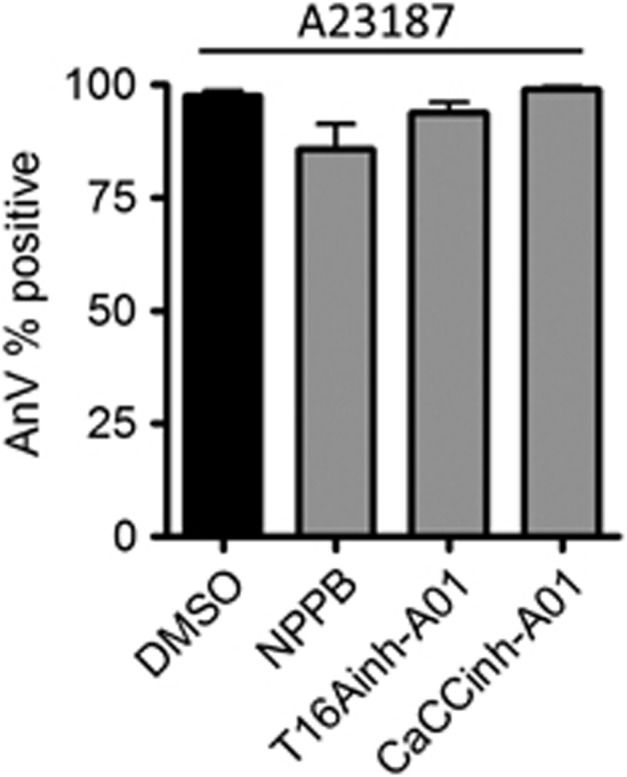
Chloride channels are not required for Ca^2+^-dependent PS exposure. Washed platelets were stimulated with the Ca^2+^ ionophore, A23187 (10 *μ*M), and annexin V (AnV) binding was monitored (*n*=4). Histograms depict mean±S.E.M.

In contrast to their inhibition of thrombin-plus-CRP-induced PS exposure, the Cl^−^ channel blockers did not substantially inhibit PS exposure induced by the Ca^2+^ ionophore, A23187 (Figure 3). This suggests that Cl^−^ channels may regulate Ca^2+^ signalling rather than the exposure of PS in response to a sustained increase in intracellular Ca^2+^.

**Figure 4:**
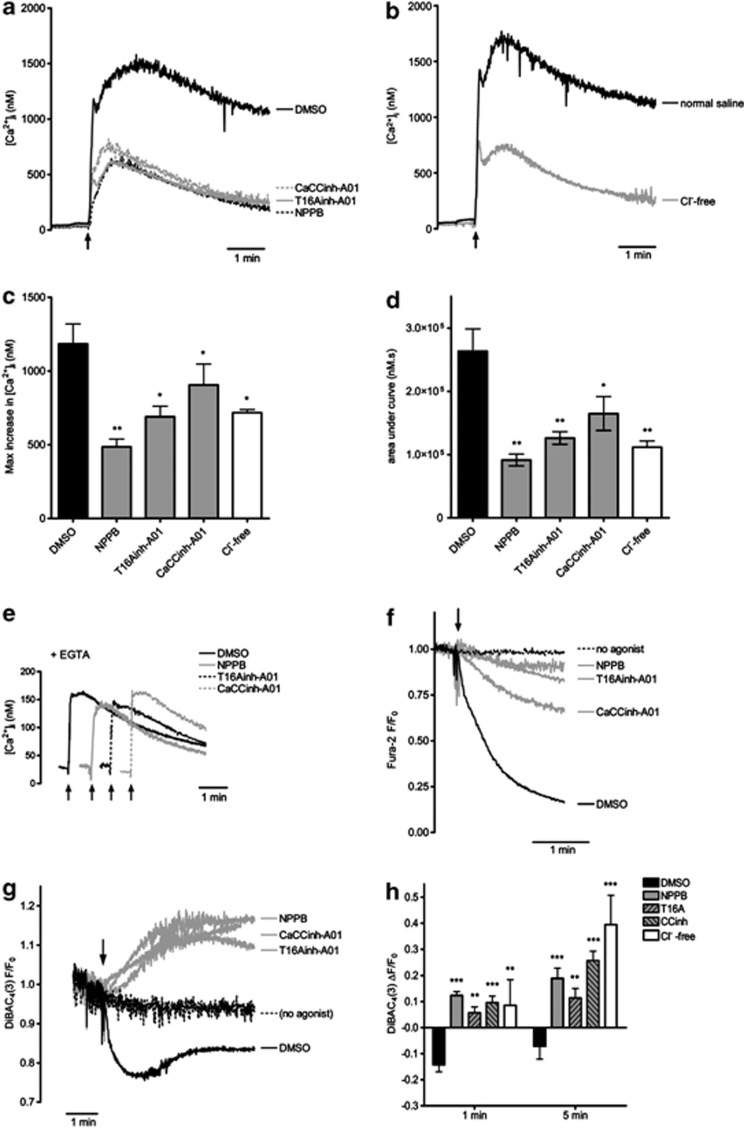
Chloride channels mediate platelet membrane hyperpolarization and maintain Ca^2+^ entry. Intracellular Ca^2+^ concentration was monitored in fura-2-loaded human platelets (**a**–**e**). Platelets were stimulated with thrombin-plus-CRP as indicated by the arrows. Traces are representative of five independent experiments. The maximum increase in [Ca^2+^]i above basal and the area under the curve are quantified in (**c**) and (**d**), respectively (*n*=5; *P<0.05; **P<0.01). (**a**, **c**) Platelets were treated with the indicated chloride channel blockers, or equivalent volume of DMSO as control, for 5 min before stimulation. (**b**, **d**) Platelets were suspended in normal NaCl-based buffer (–) or in Na-gluconate-based buffer (Cl^−^ free). (**e**) Platelets were stimulated in the absence of extracellular Ca^2+^ (1 mM EGTA added). (**f**) Mn^2+^ entry into fura-2-loaded platelets and quench of fura-2 fluorescence was used to monitor divalent cation entry. (**g**, **h**) Platelets were stimulated with thrombin and CRP in the presence of DiBAC_4_(3) in order to monitor membrane potential. Representative traces are shown in (**g**) and the mean changes in fluorescence (±S.E.M.) at 1 and 5 min after stimulation are given in (**h**) (*n*=6; *P<0.05; **P<0.01; ***P<0.001)

Platelet stimulation with thrombin-plus-CRP rapidly triggers a sustained increase in [Ca^2+^]_i_. This was substantially inhibited by the Cl^−^ channel blockers (Figure 4a). Agonist-induced Ca^2+^ signalling in Cl^−^-free medium was also substantially reduced (Figure 4b). Both the rapid peak increase and the area under the curve (AUC) were inhibited (Figures 4c and d). Agonist-induced Ca^2+^ signals are controlled by multiple pathways including Ca^2+^ release from intracellular Ca^2+^ stores and Ca^2+^ entry through Ca^2+^ channels.^19^ Cl^−^ channel blockers had no effect on thrombin-plus-CRP-induced Ca^2+^ signalling when EGTA was added to chelate extracellular Ca^2+^, indicating that intracellular Ca^2+^ release is unaffected (Figure 4e). In contrast, divalent cation entry, as monitored by Mn^2+^ quench, was substantially inhibited (Figure 4f), suggesting that Cl^−^ fluxes regulate Ca^2+^ entry.

Several platelet Ca^2+^ entry channels have been described including store-operated Ca^2+^ entry, involving Orai1,^20, 21^ and TRPC-dependent, store-independent Ca^2+^ entry.^22, 23^ Electrophysiological recordings have demonstrated that Orai1 and TRPCs are sensitive to changes in membrane potential.^24, 25^ Depolarization reduces Ca^2+^ entry through these channels, whereas hyperpolarization promotes Ca^2+^ entry. Cl^−^ fluxes are likely to affect the membrane potential. We monitored membrane potential by using the potential-sensitive fluorescent dye, DiBAC_4_(3), which shows a decrease in fluorescence upon hyperpolarization from the resting potential, whereas depolarization gives an increase in fluorescence.^26^ When platelets were stimulated with thrombin-plus-CRP, DiBAC_4_(3) fluorescence initially decreased, indicating hyperpolarization, followed by a small, gradual increase in fluorescence (Figure 4g). When Cl^−^ channels were blocked the early hyperpolarization was completely lost (Figures 4g and h), and an underlying depolarization was revealed. The same effect was seen in Cl^−^-free medium (Figure 4h). These data indicate that the early hyperpolarization is dependent on Cl^−^ entry through Cl^−^ channels.

## Discussion

Although Ano6 is critically involved in Ca^2+^-dependent PS exposure,^3, 27^ how it does this is not known. Several groups have shown that Ano6 can act as a Cl^−^ channel in a range of cells.^7, 14^ If Ano6 is acting as a Cl^−^ channel in platelets, then Cl^−^ conductance might somehow regulate Ca^2+^-dependent PS exposure. In this study we have tested whether Cl^−^ channels (whether comprising Ano6 or other proteins) can regulate platelet PS exposure. We conclude that although Cl^−^ channels are not required for platelets to expose PS in response to increased [Ca^2+^]_i_, they have a novel role in supporting platelet PS exposure by promoting Ca^2+^ entry, resulting in more rapid thrombin generation.

We propose that Cl^−^ entry through Cl^−^ channels is essential for platelet hyperpolarization. This maintains the driving force for Ca^2+^ entry through plasma membrane Ca^2+^ channels, leading to a sustained increase in [Ca^2+^]_i_. This is necessary to trigger full PS exposure and promote thrombin generation. In the absence of Cl^−^-dependent hyperpolarization, agonist stimulation leads to depolarization, probably due to Na^+^ and Ca^2+^ entry. This reduces further Ca^2+^ entry, limits PS exposure and slows the rate of thrombin generation. In this way, Cl^−^ entry indirectly regulates PS exposure. The effect of preventing Cl^−^ entry is specific to Ca^2+^ entry as Ca^2+^ release from intracellular stores was not affected.

The molecular identities of platelet Cl^−^ channels involved are unknown. Ano6 remains a possibility if, as shown in some cells, Ano6 is a component of the VSOR Cl^−^ channel.^7^ Conversely, it has been proposed that Ano6 is in fact a small conductance cation channel,^27^ in which case the identity of the platelet Cl^−^ channel remains to be discovered.

Importantly, our data suggest that PS scrambling activity does not directly require Cl^−^ entry. Although our data show that Cl^−^ channels are important in platelet PS exposure, blocking Cl^−^ conductance does not completely abolish agonist-induced PS exposure and platelet-dependent thrombin generation and has no effect on A23187-induced PS exposure. A similar conclusion has recently been reached in lymphocytes by Kmit *et al.*,^14^ who found that Cl^−^ channel blockers had no effect on ionomycin-induced PS exposure. This contrasts with the loss-of-function mutations in TMEM16F and absent Ca^2+^-dependent PS exposure in Scott syndrome patients. Importantly, this implies that the role of Ano6 in Ca^2+^-dependent PS exposure in platelets and lymphocytes is likely to be different to any putative function of Ano6 as a Cl^−^ channel.

## Materials and Methods

### Materials

A23187, NPPB, DIDS and T16Ainh-A01 were from Tocris (Bristol, UK). CaCCinh-A01 was from Calbiochem (Nottingham, UK). Fura-2 AM (acetoxymethylester) was from Teflabs (Austin, TX, USA). FITC-annexin V was from Abcam (Cambridge, UK). FITC-confjugated anti-CD62P antibody and FITC-PAC-1 antibody were from BD Biosciences (Oxford, UK). DiBAC_4_(3) was from Life Technologies (Paisley, UK). Cross-linked collagen-related peptide (CRP) was from Prof. Richard Farndale (Department of Biochemistry, University of Cambridge, UK). Tissue factor (Dade Innovin) was from Seimens Healthcare (Camberley, UK). The fluorescent thrombin substrate, Z-Gly-Gly-Arg-AMC HCl, was from Bachem (Weil am Rhein, Germany). The thrombin calibrator was from Diagnostica Stago (Theale, UK). All other reagents were from Sigma (Poole, UK), and were of analytical grade.

### Platelet preparation

Blood was obtained from healthy drug-free volunteers with approval from the local Research Ethics Committee of the University of Bristol, UK; informed, written consent was obtained in accordance with the Declaration of Helsinki. All donors reported that they had not taken any drugs known to affect platelet function (e.g., aspirin) for 10 days before donation. Fura-2-loaded human platelets were prepared essentially as previously described.^19^ Platelets were resuspended in modified Tyrodes-HEPES (135 mM NaCl, 3 mM KCl, 12 mM 10 mM Hepes, 5 mM glucose, 1 mM MgCl_2_ and 0.02 U/ml apyrase (grade VII), pH 7.3) to a density of 1 × 10^8^/ml. Unless indicated otherwise, all experiments were performed in the presence of 1 mM extracellular CaCl_2_.

### Thrombin generation

Thrombin generation was monitored in normalized PRP (1.5 × 10^8^ platelets/ml). PRP was stimulated with CRP for 5 min. Thrombin generation was triggered with tissue factor/CaCl_2_ cleavage of the substrate Z-Gly-Gly-Arg-AMC HCl measured by the thrombogram method in a Fluoroskan Ascent microplate reader at 37°C. Thrombin concentrations were determined by using a Thrombin Calibrator (Stago) following correction for the inner filter effect and substrate consumption as described by Hemker *et al.*^15^

### Flow cytometry

Annexin V-FITC was used to detect surface PS exposure. FITC-conjugated anti-CD62P antibody was used to monitor *α*-granule secretion and FITC-conjugated PAC-1 antibody was used to monitor *α*_IIb_*β*_3_ activation. Platelets (5 × 10^7^/ml) were stimulated in the presence of CaCl_2_ (1 mM) for 10 min then diluted in Tyrode's-HEPES buffer containing 2 mM CaCl_2_ and analysed immediately. Fluorescence was detected by flow cytometry, with platelets gated by their forward and side scatter profile Analysis of 20 000 platelets was performed using a Becton Dickinson FACSCalibur (Oxford, UK). Data were analysed using WinMDI version 2.8 (The Scripps Research Institute, San Diego, CA, USA).

### Calcium measurements

Fura-2 fluorescence from magnetically stirred cuvettes was monitored at 37°C using a Hitachi F-4500 (Hitachi High-Technologies, Maidenhead, UK) spectrofluorimeter with fluorescence excitation made at 340 and 380 nm, and emission at 510 nm. The ratio of emissions (*F*_340_:*F*_380_) was calibrated in terms of [Ca^2+^]_i_.^28^ Calibrations were also performed in the presence of Cl^−^ channel blockers to account for any effects on fura-2 fluorescence.

### Mn^2+^ entry

Fura-2 fluorescence was excited at the isosbestic wavelength (359 nm) and emission detected at 510 nm. Autofluorescence was subtracted and fluorescence was expressed relative to the initial fluorescence (*F/F*_0_). Mn^2+^ entry was detected as a quench of fura-2 fluorescence.

### Membrane potential measurement

Platelet membrane potential was monitored with the potential-sensitive fluorescent dye, DiBAC_4_(3). Platelets were incubated with DiBAC_4_(3) (100 nM; 30 min) prior to stimulation. Fluorescence was excited at 490 nm and emission detected at 590 nm. Fluorescence was expressed relative to the initial value (F/F_0_).

### Data analyses

Mean data are presented with standard errors; the number of replicates of each experiment, representing independent experiments from platelet preparations from different donors is reported in the figure legends. Data were analysed by one-way ANOVA, with Dunnett's test for multiple comparisons. *P*<0.05 was considered statistically significant.

## Abbreviations

Ano6: Anoctamin 6
AnV: annexin V
[Ca^2+^]_i_: intracellular calcium concentration
CRP: cross-linked collagen-related peptide
DMSO: dimethyl sulfoxide
FITC: fluorescein isothiocyanate
PRP: platelet-rich plasma
PS: phosphatidylserine
